# Identification and functional analysis of the *DOF* gene family in *Populus simonii*: implications for development and stress response

**DOI:** 10.3389/fpls.2024.1412175

**Published:** 2024-05-08

**Authors:** Kewei Cai, Xiaoyu Xie, Lu Han, Junbo Chen, Jinwang Zhang, Hongtao Yuan, Jiajia Shen, Yishuang Ren, Xiyang Zhao

**Affiliations:** ^1^ Jilin Provincial Key Laboratory of Tree and Grass Genetics and Breeding, College of Forestry and Grassland Science, Jilin Agricultural University, Changchun, China; ^2^ State Key Laboratory of Tree Genetics and Breeding, Northeast Forestry University, Harbin, China; ^3^ The Forest Tree Genetics and Breeding Laboratory, Tongliao Forestry and Grassland Science Research Institute, Tongliao, China

**Keywords:** *Populus simonii*, DOF family, phylogenetic analysis, chromosome localization, expression pattern

## Abstract

**Background:**

Populus simonii, a notable native tree species in northern China, demonstrates impressive resistance to stress, broad adaptability, and exceptional hybridization potential. DOF family is a class of specific transcription factors that only exist in plants, widely participating in plant growth and development, and also playing an important role in abiotic stress response. To date, there have been no reported studies on the DOF gene family in P. simonii, and the expression levels of this gene family in different tissues of poplar, as well as its expression patterns under cold, heat, and other stress conditions, remain unclear.

**Methods:**

In this study, DOF gene family were identified from the P. simonii genome, and various bioinformatics data on the DOF gene family, gene structure, gene distribution, promoters and regulatory networks were analyzed. Quantitative real time PCR technology was used to verify the expression patterns of the DOF gene family in different poplar tissues.

**Results:**

This research initially pinpointed 41 PSDOF genes in P. simonii genome. The chromosomal localization results revealed that the PSDOF genes is unevenly distributed among 19 chromosomes, with the highest number of genes located on chromosomes 4, 5, and 11. A phylogenetic tree was constructed based on the homology between Arabidopsis thaliana and P. simonii, dividing the 41 PSDOF genes into seven subgroups. The expression patterns of PSDOF genes indicated that specific genes are consistently upregulated in various tissues and under different stress conditions, suggesting their pivotal involvement in both plant development and response to stress. Notably, PSDOF35 and PSDOF28 serve as pivotal hubs in the interaction network, playing a unique role in coordinating with other genes within the family.

**Conclusion:**

The analysis enhances our comprehension of the functions of the DOF gene family in tissue development and stress responses within P. simonii. These findings provide a foundation for future exploration into the biological roles of DOF gene family.

## 1 Introduction

The DNA binding with one finger (*DOF*) transcription factors are a group of specific transcription factors exclusively present in plants, encompassing gymnosperms, angiosperms, and certain lower algae. *DOF* transcription factors belong to a subfamily within the zinc finger protein family, characterized by the presence of oligomerization sites, nuclear localization signals, and two principal domains encompassing a total of 200 – 400 amino acids (Zou and Sun, 2023). One of the domains is called the N-terminal DNA-binding domain, also known as the DOF domain, which exhibits a highly conserved C_2_-C_2_ single zinc finger structure composed of the CX_2_CX_21_CX_2_C motifs. This single zinc finger structure endows it with unique DNA recognition abilities, especially the ability to specifically recognize the AAAG/CTTT functional element in plant promoter sequences. Moreover, there are four absolutely conserved Cys residues and a Zn^+^ covalently bound in this single zinc finger structure. Zn^+^ and Cys residues are necessary for the DOF protein to maintain its activity (Khan et al., 2023). The other domain is the transcriptional regulatory domain located at the C-terminal of the DOF protein. Its amino acid sequence is relatively variable and lacks conservatism, which leads to the functional diversity of the DOF protein in plants (Noguero et al., 2013; Gupta et al., 2015). The distinctive structures and functionalities of the *DOF* gene family allow it to bind to DNA and interact with regulatory elements, participate in various physiological and biochemical processes in plants, and play a crucial role in regulating gene expression at all stages of plant growth and development, as well as in resisting stress responses (Zou et al., 2019; Ruta et al., 2020).

Based on the findings of genome-wide analysis, *DOF* genes are abundantly present across the genomes of diverse plants. For instance, in model plants, we observed the presence of 36 *DOF* genes in *Arabidopsis thaliana*, whereas 30 members of the *DOF* family were identified in *Oryza sativa* (Lijavetzky et al., 2003). *DOF* family members have also been discovered in ornamental plants, including *Chrysanthemum morifolium*, where a total of 20 *DOF* genes were identified (Song et al., 2016). Furthermore, the *DOF* family is ubiquitous in numerous crop species. Specifically, 33 *DOF* family members have been identified in *Capsicum annuum* (Wu et al., 2016), while 20 members were detected in *Spinacia oleracea* (Yu et al., 2021). And, 74 family members were discovered in *Musa acuminata* (Dong et al., 2016), and 36 family members in *Cucumis sativus* (Wen et al., 2016). Moreover, the *DOF* family’s presence has been confirmed in woody plants as well. Specifically, 25 *DOF* genes have been detected in *Prunus persica* (Chen et al., 2017), while 24 members of the *DOF* family have been identified in *Prunus sibirica* (Li et al., 2022a). There are also related studies in Salicaceae plants. For example, Wang et al. identified the 41 *DOF* genes in *Populus trichocarpa* and studied the expression level of it under osmotic stress and abscisic acid stress (Wang et al., 2017). In addition, Wang et al. also identified the 44 *DOF* genes in *Populus simonii* × *Populus nigra* and elucidated the role of *DOF* genes in promoting nitrogen assimilation and utilization (Wang et al., 2022). These extensive studies indicate that *DOF* family genes are universally distributed throughout the plant kingdom, laying a solid foundation for further investigations into their diverse functions and roles. Nonetheless, there have been no reported studies on the *DOF* gene family in *P. simonii*, and the expression levels of this gene family in different tissues of poplar, as well as its expression patterns under cold, heat, and other stress conditions, remain unclear.

Numerous investigations have underscored the pivotal role of *DOF* genes across a spectrum of biological processes, spanning from plant growth and development to stress responses and gene regulation. Currently, the involvement of *DOF* family members in the developmental processes and stress tolerance of diverse plants, such as grape, tea, petunia, poplar, and *A. thaliana*, has been firmly established. In grape, a comprehensive analysis identified 25 *DOF* genes, among which *VaDOF17d* was found to notably elevate raffinose levels in callus under cold stress conditions (Wang et al., 2021). After experiencing varying degrees of drought stress, the expression levels of specific *DOF* family members in tea underwent significant changes. Notably, the expression of *CsDOF10* was significantly upregulated under moderate drought conditions, indicating its crucial role in coping with drought stress (Yu et al., 2020). In petunias, *DOF* family members participate in the reproductive development of plants. Specifically, the expression of *PiDOF14* effectively promotes the degradation of the tapetal layer, thereby inhibiting meiosis of microspore cells and ultimately resulting in a male sterility phenotype (Yue et al., 2021). In *A. thaliana*, the overexpression of *AtDOF4.1* leads to significant delays in flowering and developmental defects in reproductive organs. Simultaneously, the overexpression line of this gene exhibits reduced organs, including leaves, flowers, and stems, indicating that *AtDOF4.1* functions as a transcription suppressor (Ahmad et al., 2013). Furthermore, the overexpression of the gene *PnDOF30*, originating from *P. simonii* × *P. nigra*, in *A. thaliana* notably enhances the growth of transgenic *A. thaliana* plants when subjected to low nitrogen conditions (Wang et al., 2022).

*P. simonii*, a notable native tree species in northern China, demonstrates impressive resistance to stress, broad adaptability, and exceptional hybridization potential (Chen et al., 2013). Considering the substantial impact of *DOF* genes on growth and stress responses in various plant species, coupled with the scarcity of research on this gene family in *P. simonii*, it becomes imperative to further explore the functions and contributions of *DOF* genes within this particular species. To comprehend the unique traits and functions of these genes within *P. simonii*, we explored multiple aspects, including identifying family members, phylogenetic relationships, chromosome localization, gene structure, specific expression profiles, protein-protein interactions, and conducting qRT-PCR analysis. This study provides valuable insights for further research on the potential roles of the *DOF* gene family in the growth, development, and stress resistance of *P. simonii*.

## 2 Materials and methods

### 2.1 Identification of *DOF* genes in *P. simonii*

In order to identify the *DOF* family members within the *P. simonii* genome, we obtained the full DOF protein sequence of *A. thaliana* from the Tair database (https://www.arabidopsis.org/). Moreover, we constructed a high-quality reference genome of *P. simonii*, featuring a contig N50 of 24 Mb (Cai et al., 2023). Initially, we used the BlastP program (e-value, 1 × e^−5^) in Tbtools software (v2.080) to identify PSDOF protein sequences, employing *A. thaliana* DOF proteins as query sequences (Chen et al., 2020). From the search results, we eliminated duplicate sequences to obtain the presumptive member sequences. Subsequently, to further validate the candidate protein sequences, we utilized the NCBI “batch Web CD-Search Tool” (https://www.ncbi.nlm.nih.gov/Structure/bwrpsb/bwrpsb.cgi). This tool can be used to detect and confirm whether each candidate protein is a bona fide DOF protein based on the presence of conserved domains. Finally, we employed the online tools SMART (http://smart.embl-heidelberg.de/smart/set_mode.cgi) and Pfam (http://pfam-legacy.xfam.org/) to perform rigorous verification of the conserved domains within our search results. Through this extensive verification process, we were able to identify the potential *PSDOF* genes. Additionally, using Tbtools software, we performed comprehensive biochemical characterization of each PSDOF protein, analyzing important parameters including amino acid composition, molecular weight, and instability index.

### 2.2 Phylogenetic analyses of *DOF* genes in *P. simonii*

To explore the evolutionary relationships among PSDOF proteins, we conducted an extensive analysis utilizing the candidate protein sequences. Initially, we conducted multiple sequence alignment using the Muscle program within MEGA software (v11.0), following its default parameters (Tamura et al., 2021). Next, phylogenetic trees were constructed in MEGA using the neighbor-joining (NJ) method. To improve the tree topologies’ dependability and consistency, a bootstrapping process involving 1000 iterations was executed. For better visual understanding and analysis of phylogenetic relationships, the ultimate phylogenetic tree of the *PSDOF* genes underwent refinement and annotation via the Interactive Tree of Life (iTOL) web tool (https://itol.embl.de/).

### 2.3 Chromosomal localization and synteny analysis of *PSDOF* genes

The Tbtools software was utilized to identify the chromosomal locations of *DOF* genes on 19 chromosomes in *P. simonii*, employing General Feature Format (GFF) data for visual representation. Following this, utilizing the locational data of genes on chromosomes, the *PSDOF* genes were methodically rebranded as *PSDOF1* to *PSDOF41*. For an in-depth examination of *PSDOF* genes’ synteny within *P. simonii*, pairs of syntenic genes were pinpointed using McscanX analysis. Subsequently, the synteny ircus plot produced was meticulously developed using Tbtools software’s “Advanced Circos” program. Furthermore, Tbtools software’s “One-step MCScanX” program enabled the syntenic examination of *DOF* genes in *P. simonii*, *P. trichocarpa*, and *A. thaliana*. The results of this study were visually depicted through the “Multiple Synteny Plot” program of Tbtools software.

### 2.4 Gene structure, conserved motif and promoter cis-elements analysis

The “Gene Structure View” program of Tbtools software was employed to visually depict the exon/intron arrangement of *PSDOF* genes, utilizing genomic structural data along with gene identification information. Additionally, a web-based application called “Multiple Em for Motif Elicitation” (MEME v5.5.3), available at http://memesuite.org/tools/meme, was utilized to examine the preserved motif patterns in proteins produced by PSDOFs.

Furthermore, the promoter region sequences of *PSDOF* genes, covering a 2000 base pair area before the translational start site (ATG), were isolated from the *P. simonii* genome. Foreseeing cis-elements in these promoters was made easier using the PlantCARE tool, available at https://bioinformatics.psb.ugent.be/webtools/plantcare/html/. Following this, Tbtools software was utilized to graphically depict these cis-elements.

### 2.5 Expression analysis and interaction network construction of *PSDOF* genes

RNA sequencing was conducted on various tissues, including terminal buds (NT), axillary buds (NB), leaves (NL), stems (NS), phloem (NP), and roots (NR) of *P. simonii*, to assess the expression level of *PSDOF* genes, more details about the plant materials and sample collection can be found in Cai et al., 2023. Subsequently, the expression levels were graphically depicted using heat maps generated with Tbtools software. In order to delve deeper into how *PSDOF* genes are expressed under various stress scenarios, the data gathered included information from heat, cold, and salt stress treatments. Subsequently, the data underwent normalization and was employed to create an expression heatmap, effectively depicting the transcriptional reactions of all *PSDOF* genes linked to stress scenarios.

Furthermore, to forecast the interactions between 41 PSDOF protein sequences, protein-protein interaction (PPI) networks were developed via the STRING website (https://www.string-db.org/). Subsequently, the derived network files were rendered visible through Cytoscape software (v0.9.2) (Kohl et al., 2011).

### 2.6 RNA extraction and qRT-PCR analysis

The samples’ total RNA was extracted utilizing the plant total RNA extraction kit provided by Takara (Beijing, China). In order to validate our earlier expression profile results, we randomly selected 15 genes and conducted reverse transcription-quantitative PCR (RT-qPCR) analysis using specifically designed primers. The Tbtools software was employed to create customized primers for each gene, using *Actin* as the benchmark internal gene (Regier and Frey, 2010) (**Supplementary Table S1**). The ABI 7500 RT PCR system was utilized in our qRT-PCR analysis. The comparative expression levels of the chosen genes were determined through the 2^-ΔΔCt^ method (Pfaffl, 2001). For the purpose of maintaining consistency and dependability, three biological duplicates were employed in the analysis of gene expression.

## 3 Results

### 3.1 Identification of *PSDOF* genes and its physicochemical properties

Using the protein sequences of *A. thaliana* as a reference, we successfully identified 41 members of the *DOF* family in *P. simonii* through alignment and screening processes. Subsequently, each of the 41 *PSDOF* genes was named in sequential order based on their homologous chromosome positions, ranging from *PSDOF1* to *PSDOF41*. Delving deeper into *PSDOF* genes’ characteristics, along with other basic information, were summarized in **Supplementary Table S2**. The analysis revealed variations among the *PSDOF* genes. Specifically, the number of amino acids ranged from 159 to 504, with *PSDOF40* showed the largest count with 504 amino acids, and *PSDOF10* was the smallest with 159. The lipid amino acid index exhibited a wide range, varying from 43.58 to 65.29, whereas the predicted isoelectric point (pI) ranged from 4.76 to 9.48. Additionally, the instability index displayed significant variation, spanning from 38.85 to 72.38. Lastly, the molecular weights exhibited a range from 17.70 to 55.21 kDa, with *PSDOF1* exhibiting the lowest molecular weight of 17.70 kDa and *PSDOF41* displaying the highest at 55.21 kDa.

### 3.2 Phylogenetic analysis of *PSDOF* genes

**Figure 1:**
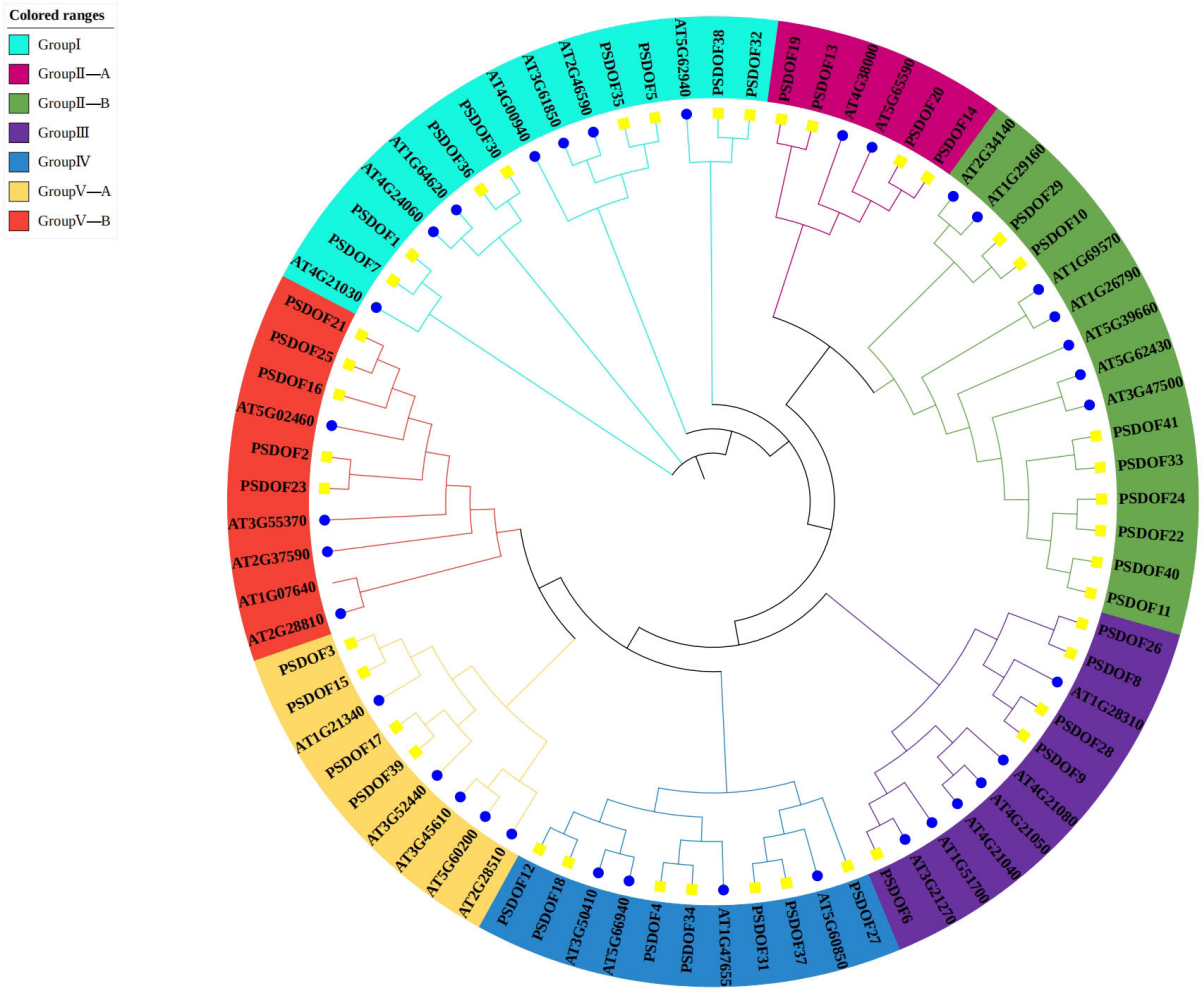
Phylogenetic relationship among *PSDOF* genes. Seven distinct subfamilies, symbolized by cyan, pink, green, purple, blue, yellow, and red arcs.

Based on the homology of DOF proteins within the phylogenetic tree, the 41 *PSDOF* genes were classified into seven distinct subfamilies, specifically designated as Group I, Group II-A, Group II-B, Group III, Group IV, Group V-A, and Group V-B (**Figure 1**). The subfamily containing the largest gene count encompassed eight members of the *DOF* family. On the other hand, the subfamily with the least number of members were Group II-A and Group V-A, both having four members from the *DOF* family.

### 3.3 Motif composition of *PSDOF* genes

**Figure 2:**
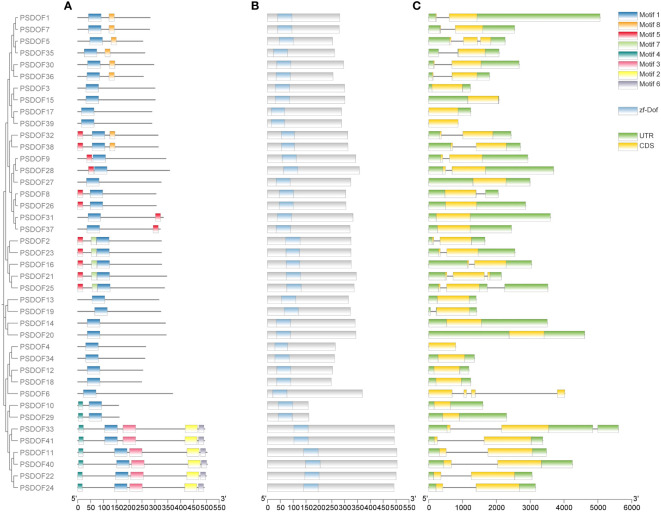
Motif, conserved domain, and gene structure analysis of *PSDOFs* family. **(A)** The arrangement of motifs in the PSDOF protein, motif 1-6 is depicted through boxes in various colors. **(B)** Conserved domain of *PSDOF* genes. **(C)** Structure of exons and introns in *PSDOF* genes. The areas of UTR, exons, and introns are depicted using green boxes, yellow boxes, and black lines, in that order.

To further explore the functional domain of PSDOF proteins, we undertook a thorough analysis of the amino acid motifs (**Figure 2A**). The majority of *PSDOF* genes are grouped together on a solitary clade, exhibiting a uniform motif composition at corresponding positions, hinting at potential similarities in their biological functionalities. However, there are significant differences in motif composition among different groups. For example, the most abundant and complex motif arrangement is observed in Group II-B, while Group II-A, Group IV, and Group V-A display the fewest number of motifs and the simplest arrangement. Moreover, particular motifs demonstrated specificity towards specific groups. For instance, motif 8 is present only in all genes of Group I, while motif 7 is detected exclusively in every gene of the Group V-B branch, thus distinguishing it from other branches.

### 3.4 Structure composition of *PSDOF* genes

In order to gain deeper insights into the local structural characteristics of the DOF domain, we conducted a comparative analysis of PSDOF protein sequences. The results observed that each gene harbors a common conserved domain zf-DOF, indicating a high degree of conservation within the *PSDOF* family domain (**Figure 2B**).

Furthermore, an exon-intron analysis was performed on *PSDOF* genes to elucidate their structural diversity (**Figure 2C**). The research identified introns in 24 genes, with most genes having only one intron, as seen in *PSDOF1* and *PSDOF7*, while a minority contained three introns, such as *PSDOF6*. Additionally, each gene exhibited varying numbers of exons, ranging from 1 to 4. For instance, *PSDOF6* possessed the highest number of exons with 4 exons, whereas others like *PSDOF5* and *PSDOF35* contained only one exon. The majority of family members typically contained two or three exons. In the same group, a considerable proportion of *PSDOF* genes exhibit similar gene structures in terms of exon count. For example, all family members in Group II-B possessed two exons except *PSDOF10* and *PSDOF29*. Notably, *PSDOF6* possesses exons and introns but lacks untranslated regions (UTRs), suggesting it may have undergone significant evolution.

### 3.5 Analysis of cis-elements in the promoter regions of *PSDOF* genes

**Figure 3:**
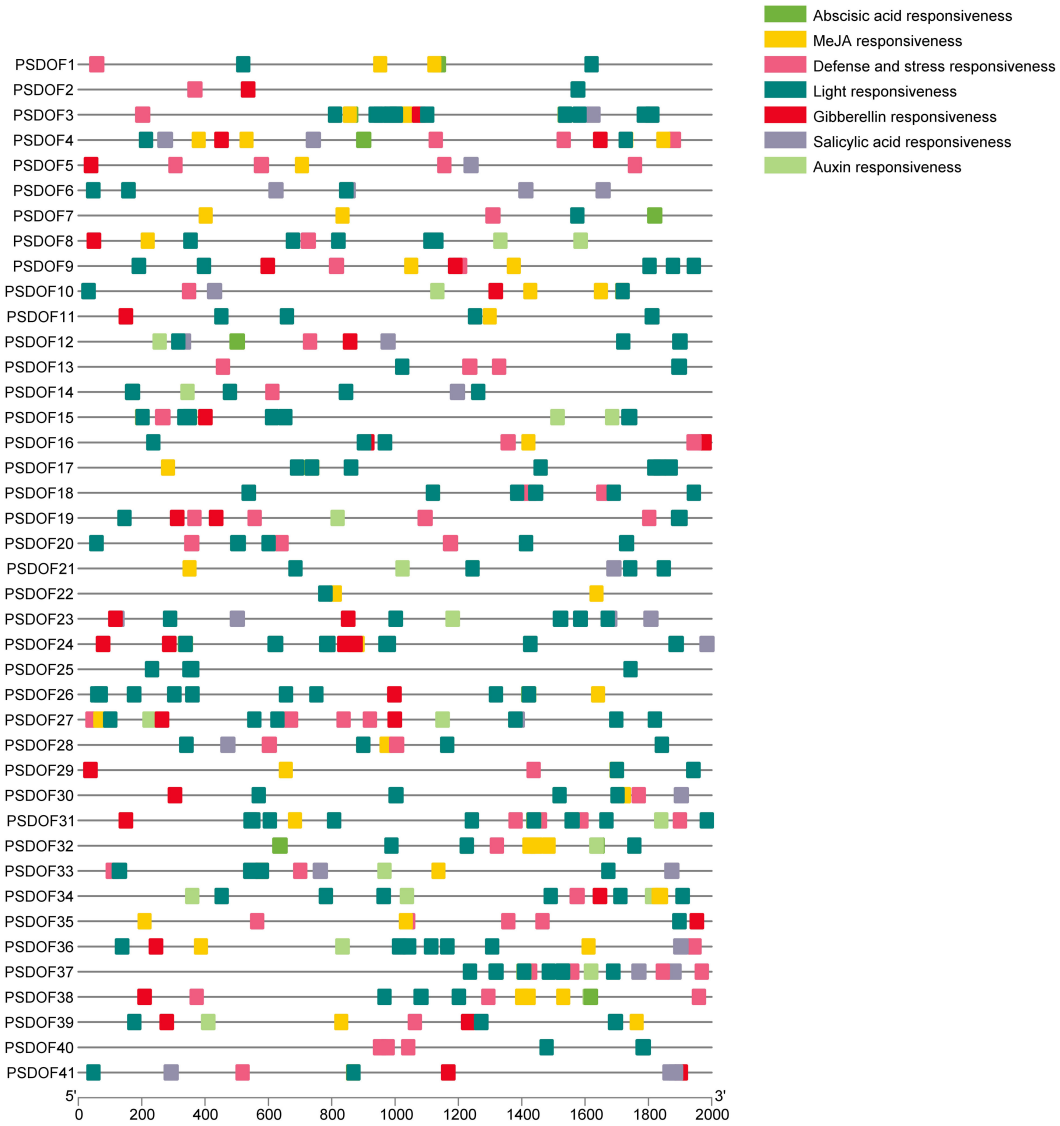
Analysis of cis-acting elements of *PSDOFs* promoter. The brightly hued squares to the right represent cis-elements, each serving distinct roles.

For exploring the biological roles of *PSDOFs*, the PlantCARE database was employed to examine cis-elements in the preceding 2 kb sequences of each gene. In the promoter region upstream, seven unique cis-elements were discovered, mainly consisting of elements responsive to growth, hormones, and abiotic stress (**Figure 3**). The element influencing plant growth encompassed responsiveness to light. Hormone response elements included responsiveness to auxin, MeJA, gibberellin, abscisic acid, and salicylic acid. Abiotic stress response elements mainly consisted of defense and stress responsiveness. The varied nature of cis-elements indicates the vital involvement of *PSDOF* genes in numerous biological functions, especially in a range of hormonal reactions and other vital biological routes.

### 3.6 Chromosome localization and synteny relationship of *PSDOF* genes

**Figure 4:**
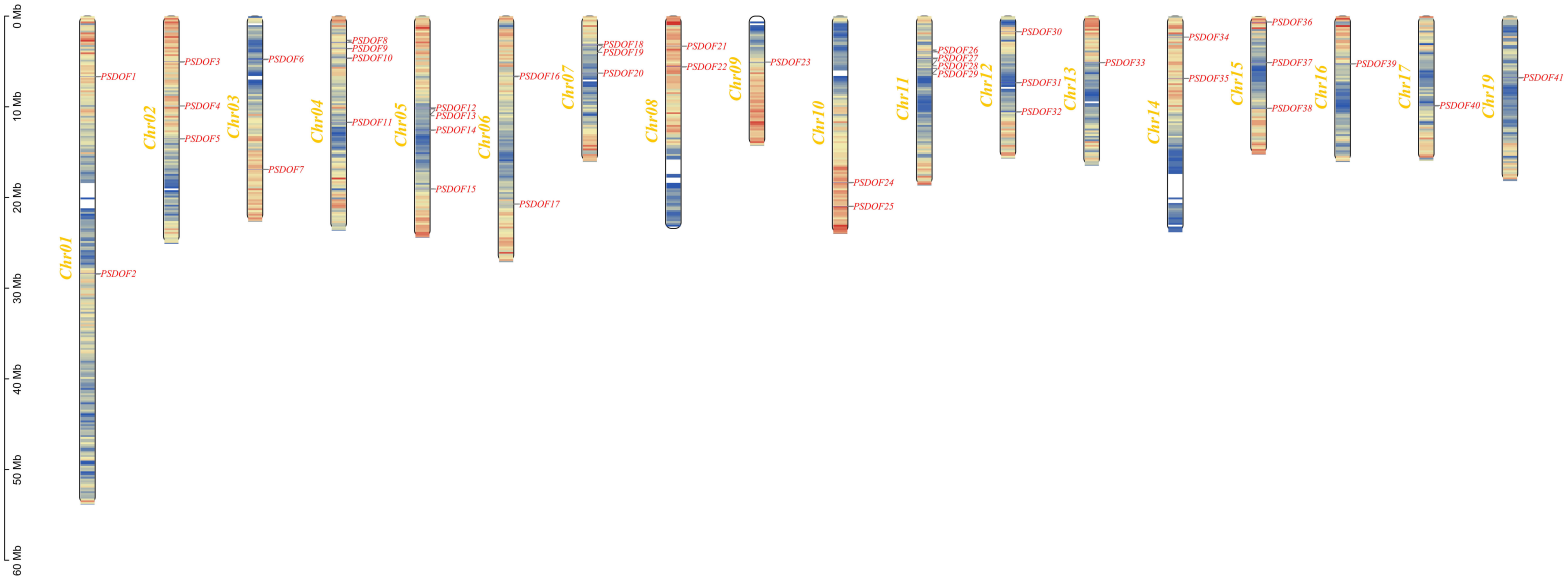
Chromosome distribution of *PSDOFs* family. Each gene’s genomic position is accurately charted on the chromosome, determined by its distinct physical coordinates. The left-side displays the chromosome count (Chr01-Chr19), while the right-side lists all the genes.

By analyzing the genomic data of *P. simonii*, we unveiled the chromosomal distribution of *PSDOF* genes, as illustrated in **Figure 4**. Among the 19 chromosomes of *P. simonii*, except for chromosome 18 where the *PSDOF* genes was not found, the number of *PSDOF* genes distributed on the remaining 18 chromosomes varies. Notably, *PSDOF* genes were most densely distributed on chromosomes 4, 5, and 11, each containing four genes. However, certain chromosomes harbored only one *PSDOF* gene, such as chromosome 9 and 16. Furthermore, the largest chromosome, chromosome 1, contained only two genes, *PSDOF1* and *PSDOF2*.

**Figure 5:**
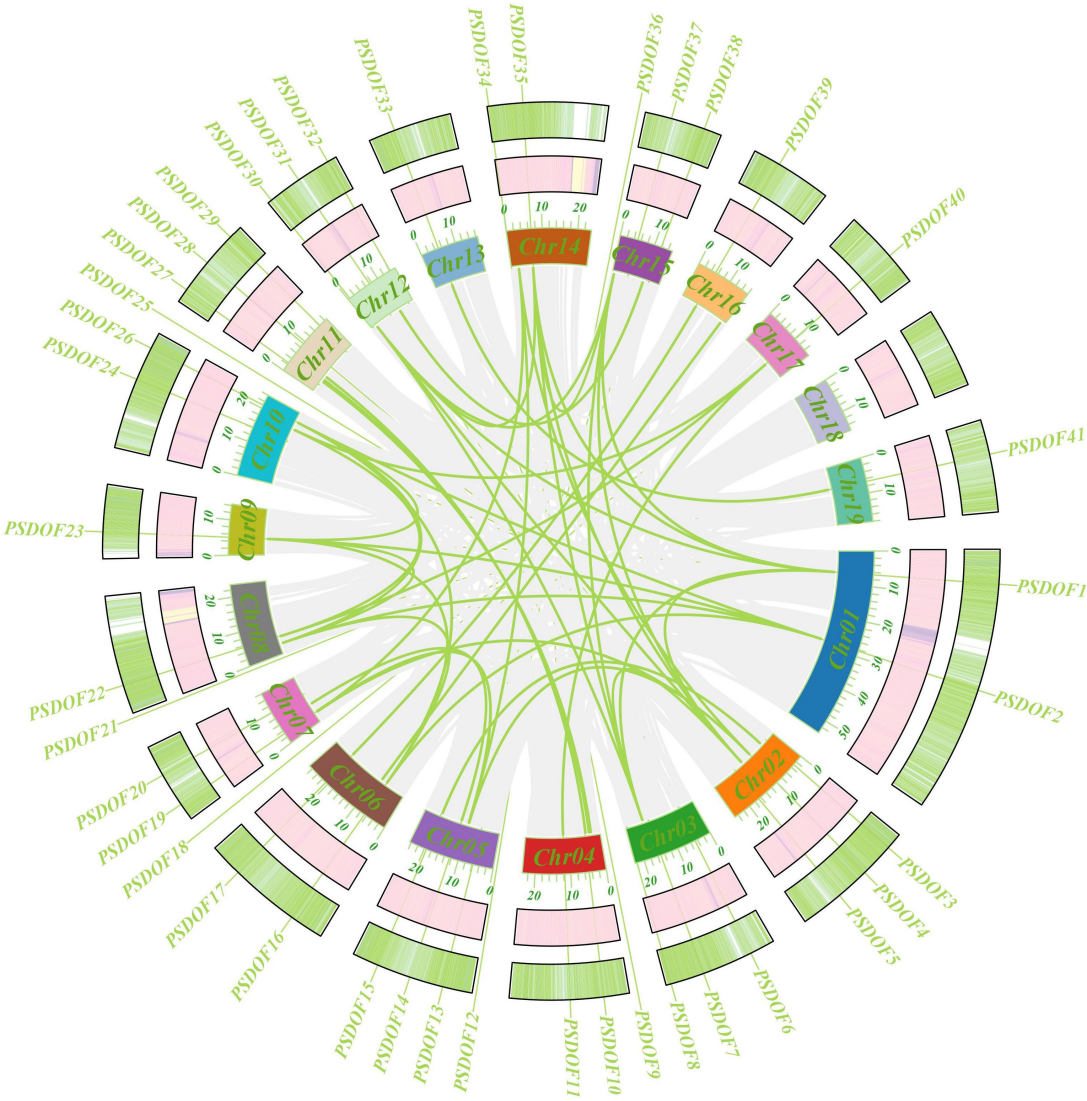
Circso diagram of *PSDOF* genes. The external to internal heat map illustrates how gene density and GC ratio are distributed along the chromosome. Syntenic segments in the *P. simonii* genome are denoted by gray lines, while green lines represent pairs of *PSDOF* genes that are collinear.

For an in-depth exploration of the evolutionary connections within the *PSDOF* gene family, synteny analysis was performed (**Figure 5**). The study uncovered 37 pairs of homologous genes within the 41 *PSDOF* genes, predominantly on chromosomes 2 and 14, with a higher prevalence of homologous gene pairs, succeeded by chromosome 8. In contrast, chromosome 13 had a relatively small count of homologous gene pairs. It’s notable that certain *PSDOF* genes show associations with multiple genes situated on different chromosomes.

### 3.7 Synteny relationships of *DOF* genes in *P. simonii* and different species

**Figure 6:**
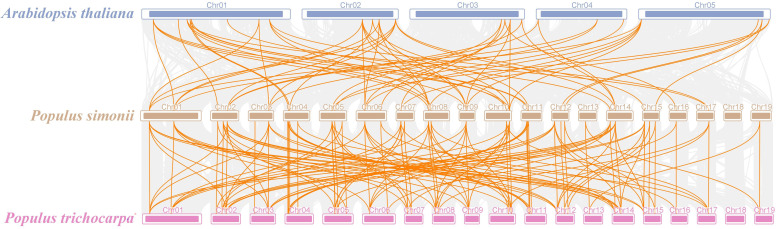
Synteny relationship of *PSDOF* genes among *P. simonii*, *A. thaliana* and *P. trichocarpa*. Orange lines indicate pairs of *DOF* genes aligned in a straight line, while gray lines show segments of *P. simonii* that are similar in structure to the genomes of both plants.

Aiming to delve more profoundly into the development of the *PSDOF* gene family, we created a comparative chart that includes *P. simonii* and two notable species, *P. trichocarpa* and *A. thaliana* (**Figure 6**). Of the 41 *PSDOF* genes, 62 gene pairs were identified as homologous to *A. thaliana*, while 119 gene pairs were found to be homologous to *P. trichocarpa*. These homologous gene pairs with *P. simonii* displayed varying distributions on each chromosome. Notably, homologous genes in *A. thaliana* and *P. trichocarpa* were predominantly concentrated on chromosome 8 of *P. simonii*, with 12 and 16 pairs, respectively. Conversely, homologous gene pairs between *A. thaliana* and *P. simonii* were dispersed on chromosome 7, 15, and 17 of *P. simonii*, with only two pairs of homologous genes. Notably, there was no distribution of homologous gene pairs on chromosome 19 of *P. simonii*.

Analogously, the distribution of homologous genes between *P. trichocarpa* and *P. simonii* exhibited variation, as there were a higher number of homologous genes located on chromosomes 1-14 and a lower number on chromosomes 16, 17, and 19. Intriguingly, the homology in *DOF* genes between *P. simonii* and *P. trichocarpa* was markedly greater compared to those with *A. thaliana*. The disparity could be ascribed to their more intimate familial ties and evolutionary background.

### 3.8 Analysis of *PSDOF* genes regulatory network

**Figure 7:**
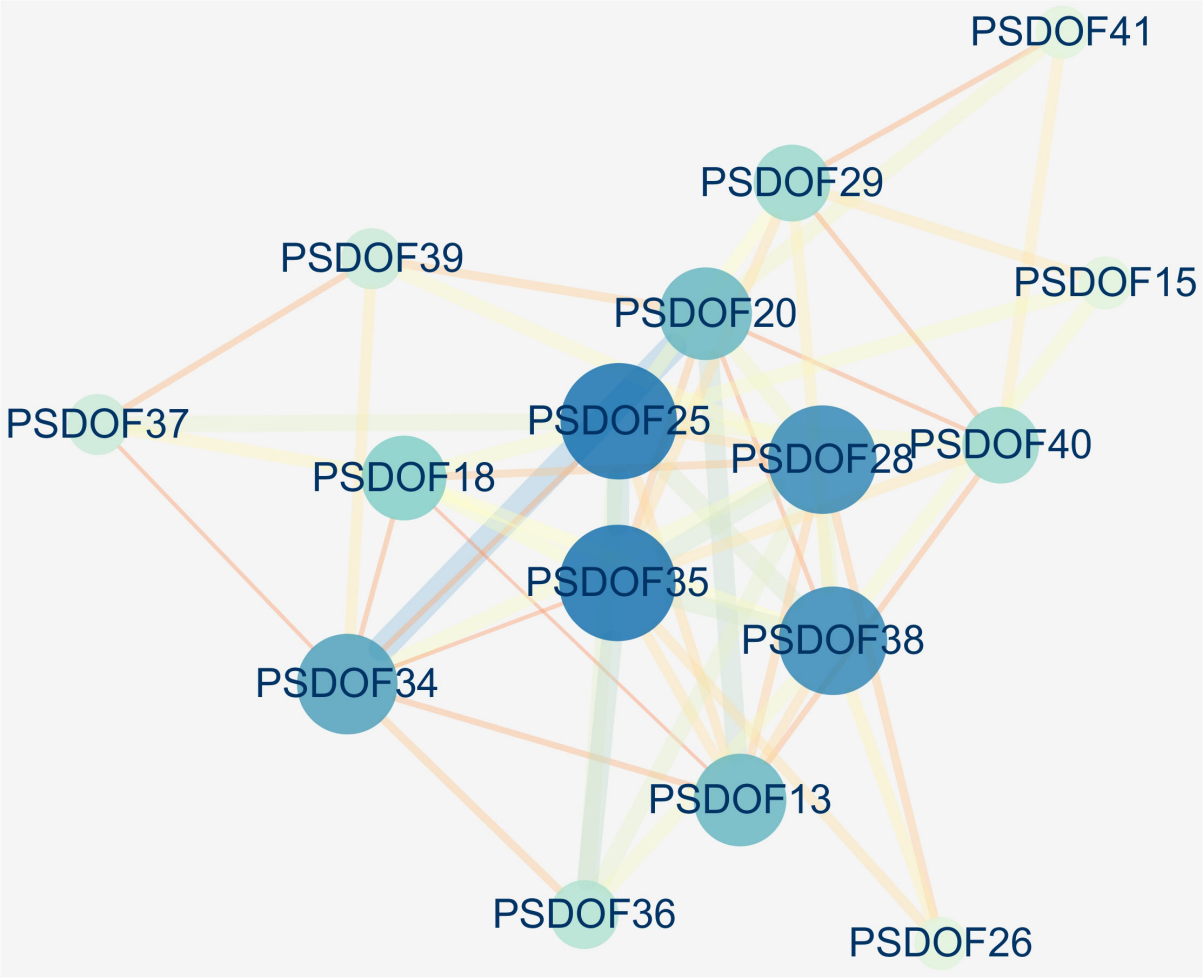
Interaction network of *PSDOF* genes. Network nodes represent proteins, while edges represent interactions between proteins in the network.

Based on the homology of *PSDOF* genes with *A. thaliana* proteins, we constructed a protein interaction network encompassing all *PSDOF* genes, aiming to gain deeper insights into the potential connections between these genes. The results, depicted in **Figure 7**, revealed that 16 out of 41 *PSDOF* genes were implicated in the construction of the gene regulatory network. Among these genes, *PSDOF25*, *PSDOF28*, *PSDOF35*, and *PSDOF38* exhibited stronger interactions with others, playing pivotal roles in the protein interaction network. As an example, *PSDOF25* functioned as a central gene, engaging with eight additional genes, such as *PSDOF29*, *PSDOF35*, and *PSDOF36*. Furthermore, *PSDOF20* also played a pivotal role in the network by engaging in interactions with seven other genes. Conversely, *PSDOF37* and *PSDOF40* exhibited limited interactions with other genes, with *PSDOF37* interacting solely with *PSDOF39* and *PSDOF40* interacting exclusively with *PSDOF41*.

### 3.9 Analysis of *PSDOF* genes expression profiles in different tissues

A thorough investigation into the functions of *DOF* genes in plant development and stress response is imperative for enhancing our comprehension of the biological roles played by these genes. For an in-depth analysis of the distinct roles of *DOF* genes in *P. simonii*, transcriptome sequencing was employed to thoroughly investigate these genes’ expression profiles in different tissues of *P. simonii*.

**Figure 8:**
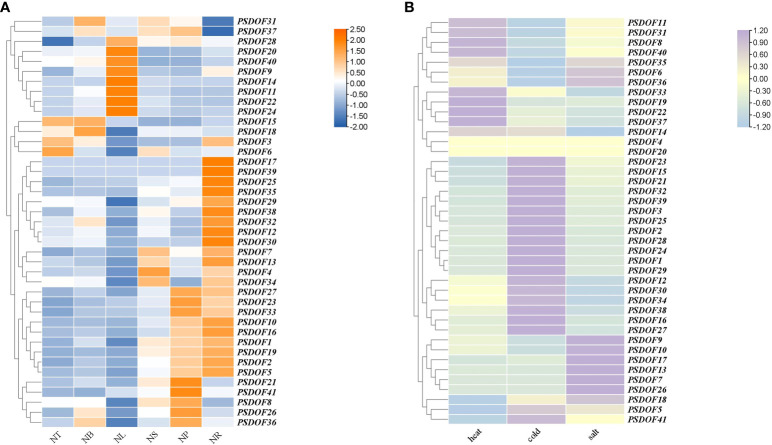
Expression patterns of *PSDOF* genes. **(A)** A thermal chart depicting *PSDOF* genes across various tissues. The shift in the color spectrum from blue to orange signifies varying degrees of expression, ranging from low to high. **(B)** Patterns of *PSDOF* genes expression under varying stress conditions. The shift in color from blue to purple symbolizes the evolution of expression levels within the spectrum, ranging from low to high.

The results demonstrated in **Figure 8A** showed that *PSDOF* genes were expressed in all tissues of *P. simonii*, but their expressions were significantly different. For example, the genes *PSDOF20*, *PSDOF14*, and *PSDOF11*, which have higher expression in the NL, have significantly lower expression in other tissues such as NS, and NP. On the other hand, genes such as *PSDOF7*, *PSDOF13*, and *PSDOF34* were highly expressed in two tissues, namely NS and NR. It was further observed that most of the genes, such as *PSDOF27*, *PSDOF23*, and *PSDOF33*, had relatively high expression in the NR and NP, low expression in the NT and NB. It is noteworthy that compared to other genes, the hub gene *PSDOF28* exhibits relatively high expression levels across various tissues, indicating its potential predominant role in the growth and development of *P. simonii*. Meanwhile, the other three hub genes such as *PSDOF25* show relatively high expression levels specifically in roots, suggesting their potentially pronounced roles in root function.

### 3.10 Analysis of *PSDOF* genes expression profiles in different stress conditions

Through the examination of *P. simonii* transcriptome data in conditions of heat, cold, and salt stress, the expression patterns of *PSDOF* genes under different stressors were extensively studied (**Figure 8B**). The results indicate that under heat stress conditions, the expression levels of eight genes including *PSDOF11*, *PSDOF31*, and *PSDOF8* are elevated, while the expression levels of the remaining members of the *PSDOF* family are relatively low. Twenty *PSDOF* genes, including *PSDOF23*, *PSDOF15*, and *PSDOF21*, among others, exhibited elevated expression levels under cold stress conditions. Additionally, the expression of genes such as *PSDOF9*, and *PSDOF17* was upregulated under salt stress conditions. Additionally, we observed that the hub gene *PSDOF35* exhibits elevated expression levels under three types of stress, suggesting its involvement in stress response. It can be found that the genes distributed on the same branch from phylogenetic tree have a certain similarity in their expression to the same stress condition. For example, the genes *PSDOF11*, *PSDOF31*, and *PSDOF8* belonged to the same branch in the evolutionary tree, and their expression under heat stress conditions was significantly higher than that under cold and salt stress conditions.

### 3.11 Quantitative reverse transcription polymerase chain reaction analysis

**Figure 9:**
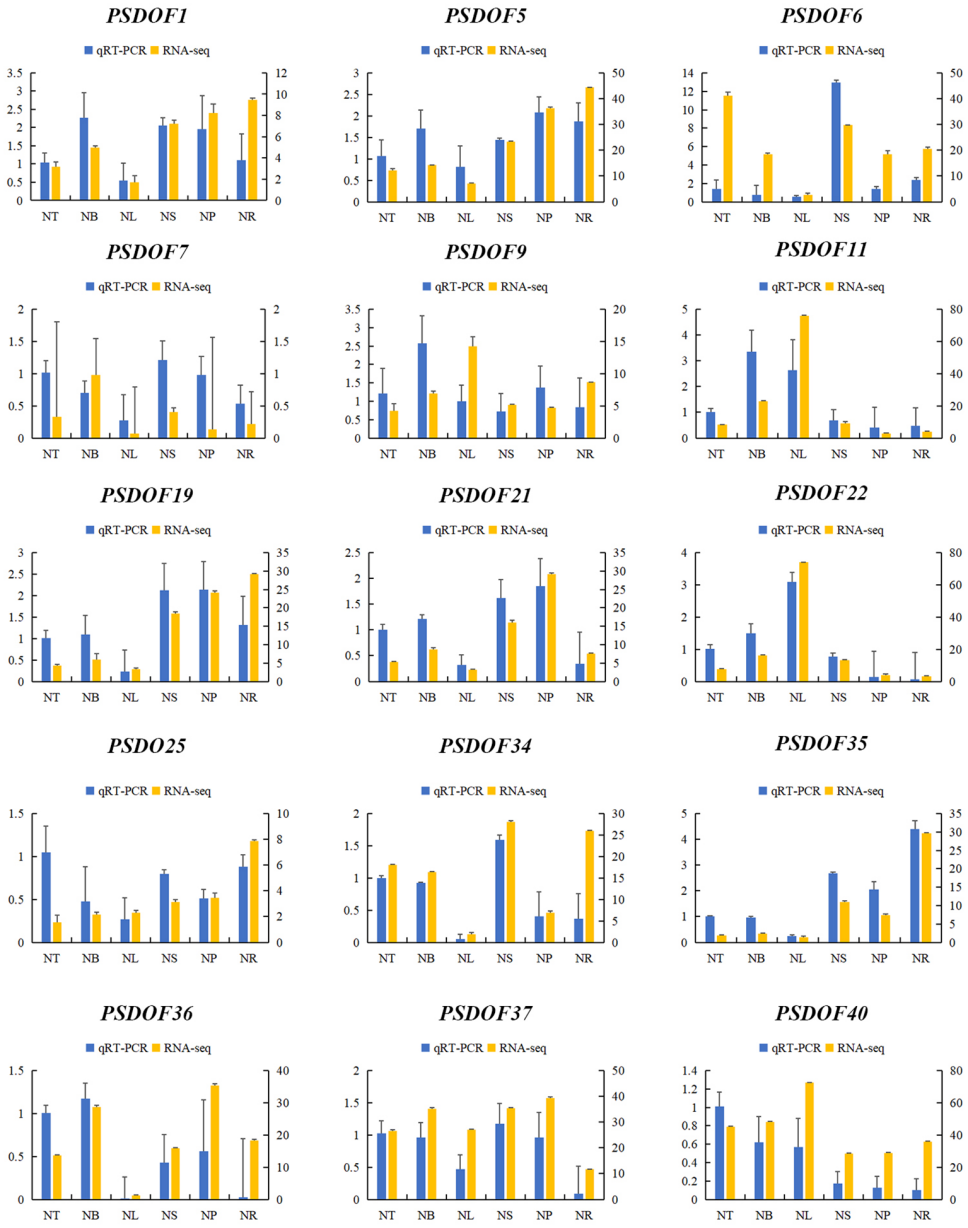
Gene expression profiles derived from RNA-seq and qRT-PCR. On the X-axis, six distinct tissues are illustrated. The expression data from qRT-PCR is displayed on the left y-axis, whereas the right y-axis illustrates the comparative expression levels of genes confirmed via RNA-seq. The error bars represent the standard error.

Fifteen *PSDOF* genes were chosen at random for qRT-PCR analysis with tailored primers, enhancing our comprehension of *PSDOF* gene expression patterns in various tissues. The way these *PSDOF* genes are expressed across various tissues aligns well with the gene expression patterns identified earlier via transcriptome sequencing, confirming the utility of RNA-seq data in evaluating these genes’ expression levels (**Figure 9**).

## 4 Discussion

The *DOF* gene family is a unique gene family in plants, playing crucial roles in plant growth, development, and adaptation to adversity. Although some members of the *DOF* family have been identified in other plant species, comprehensive genomic analysis of *DOF* genes in *P. simonii* has not been conducted, and their regulatory functions remain unclear. This study conducted a comprehensive investigation and expression analysis of the *DOF* family using genome data, providing a foundational understanding for further exploration of the development and stress responses of *P. simonii*.

Based on earlier research, the structure and number of gene families are not only related to the genome of a species, but also to the evolutionary process of a plant (Lohani et al., 2021; Waschburger et al., 2024). The research revealed 41 *DOF* genes in *P. simonii*, but the count of *DOF* genes varied across different species. When compared with reported numbers in other species, the number of *DOF* family members in *P. simonii* exceeds those reported in tea (16) (Yu et al., 2020), chickpea (37) (Nasim et al., 2016), and watermelon (36) (Zhou et al., 2020). Conversely, the count of *DOF* family members is lower than that reported in *Brassica napus* (117) (Lohani et al., 2021), cassava (45) (Zou et al., 2019), and *P. simonii* × *P. nigra* (44) (Wang et al., 2022). Additionally, the number of *DOF* genes in *P. trichocarpa* (41) (Wang et al., 2017) was similar to that in *P simonii* in this study, indicating that *DOF* genes were relatively conserved in *P simonii*.

Exploring the protein motifs within *PSDOFs* will deepen our comprehension of their unique functions in developmental and stress adaptation. In studies of motif composition, it was found that all members of the *PSDOFs* possess motif 1, indicating its potential conservatism and its determination of the functional role of *DOF* genes. Similar studies have also been conducted in sweet potato (Zhang et al., 2023) and sunflower (Song et al., 2024), where individual motifs were found to be present in all family members. This finding parallels our research results, suggesting that the motif may be a key motif within the *DOF* family, playing a crucial role in the functionality of *DOF* genes. Furthermore, the conserved motifs within the same subfamily of DOF proteins is largely consistent, suggesting that members of the DOF protein family may have similar functions in plant growth, development, and stress responses. The presence of specific motifs found exclusively among members of a particular subgroup, such as motif 8 being present in all genes of Group I, indicates the unique evolutionary significance of these motifs within that subgroup. This phenomenon has also been observed in *Brassica napus*, consistent with our research findings (Lohani et al., 2021).

The analysis of the gene structure of *PSDOFs* will help us to further explore its specific function in the process of evolution (Li et al., 2021). The analysis of the amino acid sequence revealed that the *PSDOF* gene possesses a highly conserved zf-DOF domain, suggesting a relatively conserved evolutionary trajectory for the *DOF* family in plants. The majority of *PSDOF* genes belonging to the same subgroup exhibit comparable intron-exon structures, yet a few genes demonstrate distinct and specific structures. Additionally, the number and length of introns and exons vary among genes in different branches, potentially due to extended evolutionary processes. In this study, the number of introns in all genes except *PSDOF6* was between 0 and 2, which was similar to the number of introns in maize (Chen and Cao, 2015), rice (Khan et al., 2021), pepper (Kang et al., 2016), and *Cerasus humilis* (Liu et al., 2023). This similarity suggests a relatively stable gene structure for the *PSDOF* family. Additionally, studies have reported that the presence of introns is advantageous for organismal functionality. For instance, due to alternative splicing, organisms can increase protein diversity, resulting in enhanced functional capabilities of genes (Lohani et al., 2021).

The role of cis-acting elements in gene expression is crucial, and analyzing gene promoters is key to comprehending gene expression in plants. During this study, we identified active elements, including light responsive, hormone responsive, and abiotic stress responsive elements, within *PSDOFs*. These elements play crucial roles in plant growth and development, closely resembling the cis-acting elements analyzed in wheat (Liu et al., 2020), lotus (Cao et al., 2022), and blueberry (Li et al., 2022b). These studies also indicate that the composition of *DOF* gene promoter elements is relatively conserved, and the associated *DOF* genes play roles in growth, development, and stress responses.

By examining the expression profiles of *PSDOF* genes across various tissues of *P. simonii*, we gain valuable insights into the dynamic gene expression patterns that underlie its growth and development. The findings revealed that *PSDOF* genes exhibit elevated expression levels in specific tissues of *P. simonii*, thus highlighting their crucial role in the plant’s growth and developmental processes. At the same time, a small number of *PSDOF* family members show different tissue-specific expression patterns, which means that this small number of genes may have a specific function. Similar findings have been reported in many studies as well. In *A. thaliana*, *DOF2.1 pro*, *DOF5.3 pro*, *DOF4.6 pro* gene expression in leaves an early stage plays an important role in vascular development (Gardiner et al., 2010). The expression of *OeuDOF4* gene was significantly upregulated in olive, which indicated that *OEUDOF 4* gene was involved in the development of olive leaves (Mariyam et al., 2021). Moreover, prior studies have shown the pivotal involvement of *DOF* genes across multiple stages of grape fruit development and ripening (Da Silva et al., 2016). The *DOF* genes plays a role in the production of starch in maize. When *ZmDOF36* is overexpressed, it elevates starch levels while diminishing reduced sugar and soluble sugar, aiding in the development of methods to control starch production in maize endosperm (Wu et al., 2019). Additionally, in *Brassica napus*, *BnCDF1* plays a transcriptional regulatory role in flowering time and frost resistance. Overexpression of *BnCDF1* in *A. thaliana* delays flowering time by modulating the expression patterns of CO and FT flowering time control genes, leading to significantly enhanced frost tolerance (Xu and Dai, 2016). These results indicate that *DOF* genes play irreplaceable roles in the growth and development of various plants.

*DOF* genes are crucial in controlling the non-living stress response of plants (Ma et al., 2015). In this study, we found that the expression levels of most *PSDOF* genes are significantly upregulated under stress conditions, indicating their involvement in stress adaptation. Similarly, *PSDOF* genes have been found to be induced by stress in other plants. Under salt stress, except for *PgDOF8*, the expression of other *PgDOFs* genes in pearl millet leaves was up-regulated, and the up-regulation of *PgDOF5* was the most significant (Anup et al., 2017). Under high temperature stress in passion fruit, the majority of *PeDOF* members were inducible, and the expression of *PeDOF11* was upregulated most significantly (Chen et al., 2023). In walnut, research has revealed that *JrDOF3* contributes to enhancing the heat stress response of *JrGRAS2*. Arabidopsis plants overexpressing *JrGRAS2* exhibited increased tolerance to heat stress (Yang et al., 2018). In *Gossypium hirsutum*, research has identified changes in the gene expression levels of *GhDOFD9.6* in response to salt stress. Additionally, genes such as *GhDOFA5.7*, *GhDOFA7.4*, and *GhDOFD11.3* were found to significantly respond to low-temperature stress (Li et al., 2018). Moreover, it has been hypothesized that certain *TaDOFs* in wheat serve as dynamic modulators of ROS clearance pathways, as indicated by their responsive behavior towards heavy metal stress, as reported by Liu et al. (2020). These expression analyses further confirm the roles played by *DOFs* in stress regulation. In addition, RT-qPCR analysis was conducted to confirm the RNA-seq findings, with an emphasis on the expression patterns of *PSDOF* genes.

## 5 Conclusion

This research led to the identification and categorization of 41 *DOF* genes in *P. simonii*, examining them across multiple dimensions including phylogenetic evolution, gene architecture, and evolutionary connections. The findings indicate that certain *PSDOF* family members are crucial in the growth of plant tissues and their reaction to diverse stress scenarios. Notably, *PSDOF35* and *PSDOF28* serve as pivotal hubs in the interaction network, playing a unique role in coordinating with other genes within the family. The findings of our research offer valuable insights for delving deeper into the biological roles of *DOF* genes in *P. simonii*.

## Data availability statement

The datasets presented in this study can be found in online repositories. The names of the repository/repositories and accession number(s) can be found in the article/**Supplementary Material**.

## Author contributions

KC: Writing – original draft, Writing – review & editing. XX: Data curation, Writing – review & editing. LH: Data curation, Software, Writing – review & editing. JC: Data curation, Investigation, Writing – review & editing. JZ: Formal Analysis, Methodology, Writing – review & editing. HY: Data curation, Formal Analysis, Writing – review & editing. JS: Validation, Writing – review & editing. YR: Software, Visualization, Writing – review & editing. XZ: Writing – original draft, Writing – review & editing.
